# Books

**Published:** 1883-07

**Authors:** 


### Books.

A PRACTICAL TREATISE ON IMPOTENCE, STERILITY AND ALIED DISORDERS OF THE MALE SEXUAL ORGANS. By Samuel W. Gross, A. M., M.D., Professor of the Principles of Surgery and Clinical Surgery in the Jefferson Medical College of Philadelphia, Etc., Etc. Second Edition: Henry C. Lea's Son & Co.

We doubt whether in the whole sphere of surgery there is a class of diseases whose right diagnosis and management involves so much of human happiness as the one here presented. Dr. Gross, in this admirable brochure, points out more than one novel fact for the consideration of both the scientific and general reader.

For example, the notion has long prevailed in the profession and popularly, that in almost every case of fruitless marriage the sterility is on the part of the male. The author, in the following paragraph, which we venture to quote, throws a new light on the subject:

"It is not, at all, uncommon for physicians to assume that a man who is potent and who is able to ejaculate, is capable of procreating. As a result of the omission to examine the emitted fluid, and carefully to examine the male organs, little is known of the relative frequency of sterility in the two sexes; and gynecologists, with the exception of those mentioned below, do not appear to have made any contributions to the solution of this important subject. I have been able to collect one hundred and ninety-two cases in which examination of both the husband and wife demonstrates that the former was at fault in thirty-three or in seventeen per cent. Of this number Maningham records one in thirty; Pagot, seven in eighty; Moudot one in ten; Kehrer fourteen in forty; Courty, one in ten; Noeggerath eight in fourteen; and I myself have found that the male was deficient in one example in eight. The cause of sterility was azoospermism in thirty-one and aspermatism in two. These facts show that the husband is at fault in about one case in every six, and they convey information which should be carefully weighed before the practitioner even resorts to inspection of the female organs of generation."

In this edition, again, the author takes the ground that impotence and spermatorrhœa are not mere functional diseases of the testicles, but depend upon reflex disturbances of the geneto-spinal centre, and are almost invariably induced or maintained by appreciable lesions of the prostatic portion of the urethra, which, as they are not perceived by the patient, are frequently overlooked by the physician.

This position, if established, must greatly modify the present methods of treatment. Could Dr. Gross' little hand book be read generally by the laity it would let the wind out of the inflated sails of many a genito-urinary quack.

---

Handbook of the Diagnosis and Treatment of Diseases of the Throat, Nose and Naso-Pharynx. By Carl Seiler, M.D., lecturer on Laryngoscopy at the University of Pennsylvania, Chief of the Throat Dispensary at the University Hospital, Etc., Etc.

This is emphatically the age of handbooks in science and every department of useful knowledge. There is an urgent reason for it, and those critics who write contemptuous things of the authors of hand-books, overlook a fact, which is very much to the credit of those authors for being able to see; viz: that this is an era of unsurpassed mental and physical activity in every department of industry. In order to keep oneself abreast of the world's progress, he is compelled to seize upon help in many condensed forms—in handbooks, boiled down, as it were, to the practical, from an immensity of crude verbiage and useless theory.

Busy men of all professions and trades have no time to waste in turning over cart-loads of chaff in search of a few grains of real wheat. The handbook is a necessity of the age—one of the thousand conveniences that give nimble feet and wings to progress.

This handbook of Dr. Seiler's will be welcomed by students in the interesting and too long neglected branch of practical medicine of which it treats. It is the condensed experiences of a distinguished specialist, giving in plain, unincumbered detail, just the information required by the conscientious student for the safe management of many of the most frequent and interesting diseases now known to medicine and surgery.

This is the second edition and presents voluminous additions in several important particulars to the previous edition. The illustration of instruments and topographical anatomy are numerous and minute.

